# FishShapes v1: Functionally relevant measurements of teleost shape and size on three dimensions

**DOI:** 10.1002/ecy.3829

**Published:** 2022-09-25

**Authors:** Samantha A. Price, Sarah T. Friedman, Katherine A. Corn, Olivier Larouche, Kasey Brockelsby, Anna J. Lee, Maya Nagaraj, Nick G. Bertrand, Mailee Danao, Megan C. Coyne, John R. Estrada, Rachel Friedman, Evan Hoeft, Mikayla Iwan, Dominique Gross, Jo Hsuan Kao, Brian Landry, Monica J. Linares, Carley McGlinn, Jennifer A. Nguyen, Allison G. Proffitt, Sierra Rodriguez, Maxwell R. Rupp, Erin Y. Shen, Victoria Susman, Angelly J. Tovar, Laura L. J. Vary, Katerina L. Zapfe, Peter C. Wainwright

**Affiliations:** ^1^ Department of Biological Sciences Clemson University Clemson South Carolina USA; ^2^ Department of Evolution & Ecology University of California Davis Davis California USA; ^3^ Present address: Department of Biology and Biochemistry University of Houston Houston Texas USA; ^4^ Present address: Department of Evolution, Ecology, and Behavior, School of Integrative Biology University of Illinois, Urbana‐Champaign Urbana Illinois USA; ^5^ Present address: Division of Evolutionary Biology Ludwig‐Maximilians‐Universität Munich Planegg‐Martinsried Germany; ^6^ Present address: Bureau of Reclamation Sacramento California USA; ^7^ Present address: VCA Animal Referral and Emergency Center of Arizona Mesa Arizona USA; ^8^ Independent Researcher; ^9^ Present address: College of Earth, Ocean, and Atmospheric Sciences, Oregon State University Corvallis Oregon USA; ^10^ Present address: Department of Genomics and Bioinformatics University of North Carolina at Charlotte Charlotte North Carolina USA

**Keywords:** body depth, body width, caudal peduncle depth, caudal peduncle width, ecomorphology, fishes, head depth, lower jaw length, mouth width, standard length, Teleostei

## Abstract

Teleost fishes account for 96% of all fish species and exhibit a spectacular variety of body forms. Teleost lineages range from deep bodied to elongate (e.g., eels, needlefish), laterally compressed (e.g., ribbonfish) to globular (e.g., pufferfish), and include uniquely shaped lineages such as seahorses, flatfishes, and ocean sunfishes. Adaptive body shape convergence within fishes has long been hypothesized but the nature of the relationships between fish form and ecological and environmental variables remain largely unknown at the macroevolutionary scale. To facilitate the investigation of the interacting factors influencing teleost body shape evolution we measured eight functionally relevant linear traits on adult‐sized specimens along with specimen mass. Linear measurements of standard length, maximum body depth, maximum fish width, lower jaw length, mouth width, head depth, minimum caudal peduncle depth, and minimum caudal peduncle width were taken in millimeters with calipers, or tape measures for oversized specimens. We measured these traits on a total of 16,523 specimens (1–3 specimens per species) at the Smithsonian National Museum of Natural History and took approximately 7000 person hours of data collection to complete. The data went through a three‐step error‐checking process to clean and validate the data and then species averages were calculated. We present the complete specimen data set, which encompasses approximately one‐fifth of extant teleost species diversity, spanning ~90% of teleost families and ~96% of orders. The species and family names are compatible with the taxonomy used by FishBase and the order information with the phylogenetically informed taxonomy of Betancur‐R and colleagues published in 2014. This dataset is licensed under Creative Commons CC0 1.0 Universal (CC0 1.0) but please cite this paper when using the data or a subset of it.

## CONFLICT OF INTEREST

The authors declare no conflict of interest.

## Supporting information


Data S1

## Data Availability

The complete data set is available as Supporting Information and is also available from Dryad at https://doi.org/10.5061/dryad.vt4b8gtvf.

