# Supplementary material for: FishShapes v1: Functionally relevant measurements of teleost shape and size on three dimensions
Source: Ecology. 2022 Sep 25;103(12):e3829. doi: 10.1002/ecy.3829 (PMC10078225; doi:10.1002/ecy.3829)
Supplement: Supplementary file 1 — Data S1 [file ECY-103-0-s001.zip › Data_S1/MorphometricMeasurements.pdf]

ANATOMICAL MEASUREMENTS OF FISH
A comprehensive explanation of the excel spreadsheet used for data collection.

SPECIMEN INFORMATION:

A. Specimen ID
This is the unique alphanumeric catalog number for the specimen you are measuring and it is critical it is recorded correctly. The Smithsonian will always begin with USNM. If we are measuring more than one specimen with the same ID add an underscore to the number and follow it with 1, 2, 3 etc.

B. Genus species USNM
This is the genus and species written exactly as listed on the specimen.

C. Genus species Phylogeny
Pick from the drop down menu the Latin binomial that is used within the Rabosky phylogeny for the specimen. This is only necessary when the name used in the phylogeny doesn't match the specimen label i.e. when we have synonym issues.

D. Family
Pick from the drop down menu the family that the species belongs to.

MEASUREMENTS: where possible take measurements on the left side. Remember to keep checking that your calipers are zeroed. If you are uncertain about anything ask for advice. If you think you made a mistake let someone know so we can check and correct it.

E. Total weight (g)
Be careful to choose the correct scale: max 100g, 200g, 600g and 1000g.

F. Standard length (mm)
The straight-line distance from the most anterior tip of the upper jaw to the mid-lateral posterior edge of the hypural plate (in fish with a hypural plate), or to the posterior end of the vertebral column in fish lacking hypural plates, i.e. excluding the caudal fin. This position is determined by flexing the tail up while the caudal peduncle is held down. The resultant wrinkle or caudal flexure indicates the caudal base.

G. Max body depth (mm)
The greatest depth as measured by a straight-line distance from dorsal to ventral surface of the body. The body is defined as posterior to the operculum and anterior to the caudal peduncle and be careful to not include dorsal fin.

H. Max fish width (mm)
The width of the fish measured at its maximum anywhere on the fish

I. Position of max width
Choose from the drop-down menu which region: head or body, the maximum fish width was measured on.

J. Head depth (mm)
Vertical distance from dorsal to ventral surface of the head passing through the pupil of the eye.

K. Lower Jaw Length (mm)
Length of the mandible from the anterior end of the lower jaw to the articular-quadrate jaw joint. To identify the position of the joint move the lower jaws of the fish and look for where the movement stops, in general it should be close to the end of the preopercle (when you can see it).

L. Mouth width (mm)
The width of the fish measured at the distance between the left and right articular-quadrate joints.

M. Position mouth width
Choose from the drop-down menu whether you were able to confidently identify the position of the articular-quadrate joint using the motion of the lower jaw (Found position of the articular-quadrate joint), whether it was purely inferred from the end of the preopercle (Used end of preopercle) or if it mainly inferred from anatomical diagrams (Inferred from diagrams)

N. Min caudal peduncle depth (mm)
The depth as measured by a straight-line distance from dorsal to ventral surface of the caudal peduncle at its narrowest point. The caudal peduncle is defined as between the posterior end(s) of the (dorsal and) anal fin(s) base and the caudal fin base.

O. Min Caudal peduncle width (mm)
The width of the fish measured at its minimum between the posterior end(s) of the (dorsal and) anal fin(s) base and the caudal base.

Fin spine lengths
The fins of all teleosts have fin rays, some have soft flexible fin rays while others have rigid spines on the leading edges of one or more of their fins. Spines are occasionally modified for uses other than anti-predator structures, for example the first dorsal fin spine is modified into a lure in deep sea anglerfishes (Lophiiformes), these modified spines should not be measured nor should soft fin-rays. Remember spines do not branch and are un-segmented but they can be fairly soft. If we are in any doubt we can look up the species description. If you look for a spine and can't find it enter 0 as the length and NA for the position. If you can't measure the spine because the fin is damaged leave the length cell(s) blank and make a note in column AC but if the fin is just not present in that family/species then enter NA.

P. First major dorsal spine length (mm)
The length of the first major unmodified spine in the dorsal fin and the longest spine as measured from the edge of the body to the tip of the spine.

Q. Max dorsal spine length (mm)
The length of the longest spine as measured from the edge of the body to the tip of the spine. If the first major spine is the longest spine, copy the measurement into this column and in column R note it is the 1st spine.

R. Position Max dorsal spine
Choose from the drop-down menu to give the position of the longest spine – whether it is the 1st, 2nd, 3rd etc.

S. First major anal spine length (mm)
The length of the first major unmodified spine in the anal fin as measured from the edge of the body to the tip of the spine.

T. Max anal spine length (mm)
The length of the longest spine as measured from the edge of the body to the tip of the spine. If the first major spine is the longest spine, copy the measurement into this column and in column U note it is the 1st spine.

U. Position Max anal spine
Choose from the drop-down menu to give the position of the longest spine – whether it is the 1st, 2nd, 3rd etc.

V. First major pelvic spine length (mm)
The length of the first major unmodified spine in the pelvic fin as measured from the edge of the body to the tip of the spine.

W. Max pelvic spine length (mm)
The length of the longest spine as measured from the edge of the body to the tip of the spine. If the first major spine is the longest spine, copy the measurement

into this column and in column X note it is the 1st spine.

X. Position Max pelvic spine
Choose from the drop-down menu to give the position of the longest spine – whether it is the 1st, 2nd, 3rd etc.

Y. First major pectoral fin spine length (mm) (Siluriformes – catfishes)
The length of the first major unmodified spine in the pectoral fin as measured from the edge of the body to the tip of the spine.

Z. Max pectoral fin spine length (mm) (Siluriformes – catfishes)
The length of the longest spine as measured from the edge of the body to the tip of the spine. If the first major spine is the longest spine, copy the measurement into this column and in column AA note it is the 1st spine.

AA. Position Max pectoral spine (Siluriformes – catfishes)
Choose from the drop-down menu to give the position of the longest spine – whether it is the 1st, 2nd, 3rd etc.

ADDITIONAL COLUMNS:

AB. Complete?
When you have entered all the necessary information for the specimen this will flip from WARNING INCOMPLETE to complete.

AC. Notes.
Add any additional information here. For example, if the specimen has major damage and you can’t measure the dorsal fin spines because the fin has been ripped off etc.
