# Supplementary material for: FishShapes v1: Functionally relevant measurements of teleost shape and size on three dimensions
Source: Ecology. 2022 Sep 25;103(12):e3829. doi: 10.1002/ecy.3829 (PMC10078225; doi:10.1002/ecy.3829)
Supplement: Supplementary file 1 — Data S1 [file ECY-103-0-s001.zip › MetadataS1.pdf]

Metadata S1: Samantha A. Price, Sarah T. Friedman, Katherine A. Corn, Olivier Larouche, Kasey Brockelsby, Anna J. Lee, Maya Nagaraj, Nick G. Bertrand, Mailee Danao, Megan C. Coyne, John R. Estrada, Rachel Friedman, Evan Hoeft, Mikayla Iwan, Dominique Gross, Jo Hsuan Kao, Brian, Linares Landry, Monica J. Linares, Carley McGlinn, Jennifer A. Nguyen, Allison G. Proffitt, Sierra Rodriguez, Maxwell R. Rupp, Erin Y. Shen, Victoria, Susman, Angelly J. Tovar, Laura L.J. Vary, Katerina L. Zapfe & Peter C. Wainwright. 2022. FishShapes v1: Functionally relevant measurements of teleost shape and size on three dimensions

Metadata

Class I. Data set descriptors

A. Data set identity: FishShapes v1: Functionally relevant measurements of teleost shape and size on three dimensions

B. Data set identification code:

1. DataS1/FishShapes_specimensv1.csv
2. DataS1/Read ME.pdf

C. Data set description

1. **Originator(s):**

Samantha A. Price, Department of Biological Sciences, Clemson University, Clemson SC 29634, USA. Email: sprice6@clemson.edu

Peter C. Wainwright, Department of Evolution & Ecology, University of California Davis, 1 Shields Avenue, Davis, CA 95616. Email: pcwainwright@ucdavis.edu

Abstract: Teleost fishes account for 96% of all fish species and exhibit a spectacular variety of body forms. Teleost lineages range from deep-bodied to elongate (e.g. eels, needlefish), laterally compressed (e.g. ribbonfish) to globular (e.g. pufferfish) and include uniquely shaped lineages such as seahorses, flatfishes and ocean sunfishes. Adaptive body shape convergence within fishes has long been hypothesized but the nature of the relationships between fish form and ecological and environmental variables remain largely unknown at the macroevolutionary scale. To facilitate the investigation of the interacting factors influencing teleost body shape evolution we measured 8 functionally relevant linear traits on adult-sized specimens along with specimen mass. Linear measurements of standard length, maximum body depth, maximum fish width, lower jaw length, mouth width, head depth, minimum caudal peduncle depth and minimum caudal peduncle width were taken in millimeters with calipers, or tape measures for oversized specimens. We measured these traits on a total of 16523 specimens (1-3 specimens per species) at the Smithsonian National Museum of Natural History and took approximately 7000 person hours of data collection to complete. The data went through a three-step error-checking process to clean and validate the data and then species averages were calculated. We present the complete specimen dataset, which encompasses approximately one fifth of extant teleost species diversity, spanning ~90% of teleost

families and ~96% of orders. The species and family names are compatible with the taxonomy used by FishBase and the order information with the phylogenetically informed taxonomy of Betancur-R and colleagues published in 2014. This dataset is licensed under Creative Commons CC0 1.0 Universal (CC0 1.0) but please cite this paper when using the data or a subset of it.

D. Key words: Body depth, Body width, Caudal peduncle depth, Caudal peduncle width, Ecomorphology, Fishes, Head depth, Lower jaw length, Mouth width, Standard Length, Teleostei

Class II. Research origin descriptors

A. Overall project description:

a. **Identity:** FishShapes v1: Functionally relevant shape and size linear measurements on three dimensions across teleost fishes

b. **Originator(s):**

Samantha A. Price, Department of Biological Sciences, Clemson University, Clemson SC 29634, USA. Email: sprice6@clemson.edu

Peter C. Wainwright, Department of Evolution & Ecology, University of California Davis, 1 Shields Avenue, Davis, CA 95616. Email: pcwainwright@ucdavis.edu

c. **Period of study**: Data were collected during three trips to the Smithsonian National Museum of Natural History for 2 months during the summer of 2016, 2 months during the summer of 2017 and for 3 months during the summer of 2018.

d. **Objectives:** The purpose of this research was to quantify the overall body shape morphospace of teleost fishes, providing a statistical description of the diversity of forms. This data enables the identification of the primary axes of body shape diversification across the teleost tree-of-life, as well as the patterns of body shape evolution over deep-time. Combining our data with existing ecological data also provides the means to identify instances of convergent evolution and discover whether macroevolutionary patterns are consistent with evidence from microevolutionary studies and mechanical models. These smaller scale studies reveal how body shape changes in response to ecological and environmental shifts or how particular forms maximize specific swimming and feeding performances. For example, habitat complexity (e.g. Domenici 2003), flow strength (e.g. Langerhans et al. 2007), diet (e.g. Andersson et al., 2003), position in the water column (e.g. Robinson & Parson 2002), locomotor mode (Weihs 1973), feeding mode (Rand & Lauder 1981, Lauder 1980, Wainwright et al. 2007), salinity (e.g. Sweet & Kinne 1964) and water temperature (e.g. Martin 1949) might all be expected to drive predictable patterns of body shape evolution within fishes. The dataset also enables more general evolutionary questions, such as "*Is the evolution of morphological and ecological novelty linked?*", "*How much variance in body form is explained by phylogenetic and ecological differences?*" and "*Do trait interactions and trade-offs constrain shape*

convergence?" to be answered. This dataset therefore helps to bridge between disciplines and scales to identify the major drivers of fish body shape diversity.

e. **Abstract:** Same as above. These data are not part of a larger program of study.

f. **Source(s) of funding:** National Science Foundation Division of Environmental Biology grant numbers 1556953 & 1830127 to SAP & PCW

B. **Specific subproject description**

a. **Site description**: Global trait data measured on specimens in the Smithsonian National Museum of Natural History fish collections.

b. **Experimental or sampling design:** Our aim was to broadly sample teleost body shape and taxonomic diversity and identify patterns of body shape evolution. As all our analyses would require a time-calibrated phylogeny, as species are not independent (Felsenstein 1985), we attempted to find and measure three specimens of all teleost species that had molecular data in the largest and most recent phylogeny of ray-finned fishes (Rabosky et al. 2018). We worked phylogenetically, collecting data family by family. We measured 8 linear traits that capture overall fish body shape, they are all simple to measure and are known to be ecologically and functionally relevant (once size is taken into account).

**Trait**	**Relevance**
Standard length	This is the overall measure of fish length and a standard measure of fish size.
Maximum body depth	This is the overall measure of fish depth and is related to i) swimming performance, fishes with deeper forms are generally better at turning and accelerating (e.g. Webb 1984, Domenici 2010, Langerhans & Reznick 2010) and ii) predation, with deeper bodied fish requiring larger gapes to engulf them (e.g. Brönmark & Miner 1992, Chivers et al. 2008).
Maximum fish width	This is the overall measure of fish width and is related to swimming performance (Webb 1994, Domenici 2010) and habitat choice (Helfman et al. 2009), specifically association with the substrate (Friedman et al. 2020).
Head depth	Related to prey size, some fishes with deeper heads can eat larger prey (Oliveira et al., 2010).
Minimum caudal peduncle depth	Related to swimming performance, deeper caudal peduncles are generally advantageous during fast starts (e.g. Webb 1994), while a thinner peduncle generates less drag force in lateral motion during swimming.
Minimum caudal peduncle width	Related to swimming performance, fishes with narrow tapered caudal peduncles are generally better at

	sustained straight-line swimming (e.g. Domenici 2010, Langerhans & Reznick 2010).
Lower jaw length	Related to both suction feeding and biting performance, this is the jaw closing out-lever and influences both prey type and size (Wainwright & Barton 1995, Westneat 2004, Collar et al. 2006).
Mouth width	Related to feeding performance, influencing both prey type and size (Hambright 1991, Norton & Brainerd 1993, Gatz 1979).

These traits can also be combined to generate functionally important ratios, such as fineness ratio (standard length/(maximum body depth x maximum fish width)$^{1/2}$), which describes body streamlining and is related to swimming performance (e.g., Bainbridge 1960, Walker et al. 2013).

c. Research methods

i. Instrumentation: linear measurements in millimeters were taken using a variety of electronic and dial calipers with a resolution of 0.1mm and an accuracy of +/- 0.2mm. For traits exceeding 300mm a measuring tape was used with a resolution of 1mm and an unknown accuracy. Weights of specimens were taken using a variety of scales with different weight ranges and resolutions: 100g x 0.01, 650g x 0.1, 1000g x 0.1, 2000g x 0.1 with unknown accuracy along with a scale for the heaviest fishes with range and resolution of 32000g x 1 with accuracy of +/- 50, which we borrowed from the USNM fish collections.

ii. Taxonomy and systematics: We used the tip names from the Rabosky et al. 2018 phylogeny to search the collections, checking for genus and species synonyms using the Catalog of Fishes taxonomy (Eschmeyer & Fricke 2016, 2017 & 2018) and FishBase taxonomy (Pauly & Froese 2016, 2017 & 2018). For family we used the FishBase taxonomy and for order and super-orders we used the phylogenetically informed taxonomy of Betancur-R et al. 2014. If the species name on the jar did not match the species name in the phylogeny (e.g., labeled with a junior synonym), the specimen name on the jar was also recorded in the data entry spreadsheet. Cichlidae were particularly problematic, and a wide variety of online sources were consulted in an attempt to reconcile the names on the jars and the species list from the phylogeny. We have two columns with species names: tree_name, which matches the tip labels on the phylogeny of actinopterygians built by Rabosky et al. 2018 and fishbase_name, which matches the FishBase taxonomy (as extracted on Feb 20th 2019).

iii. Specimen choice: we aimed to measure adult-sized specimens, we therefore looked for the largest and most intact specimens in the collection for each species targeted. If we were concerned that the specimen was too small to be an adult, we looked up the maximum size

for the species in FishBase and if the specimen was close to, at, or above 50% of the reported maximum length we measured it. When possible, we measured three individuals per species that had been collected at different locations (or years if it was all the same location) i.e., the three specimens were ideally from different jars and thus have different catalog numbers (specimenID).

iv. Specimen measurements: data were collected at the species level, such that each specimen (≤ 3) from the same species was measured by the same data collector. Each line of data represented a specimen, and each data collector had an individual data entry spreadsheet stored in the cloud. The spreadsheet contained 29 columns, as explained in the Read Me.pdf. The raw data were compiled and checked.

v. Specimen photograph: a lateral photograph of each fish in an ethanol-filled tank with scale bar was taken using a standard Single-Lens Reflex camera with macro lens (Nikon D90 or D7200 with 60mm lens) for subsequent analyses and data-checking purposes.

C. Project personnel:

Samantha A. Price, Department of Biological Sciences, Clemson University, Clemson SC 29634, USA & Department of Evolution & Ecology, University of California Davis, 1 Shields Avenue, Davis, CA 95616. PI

Peter C. Wainwright, Department of Evolution & Ecology, University of California Davis, 1 Shields Avenue, Davis, CA 95616. Co-PI

Sarah T. Friedman, Department of Evolution & Ecology, University of California Davis, 1 Shields Avenue, Davis, CA 95616. Ph.D. student

Katherine A. Corn, Department of Evolution & Ecology, University of California Davis, 1 Shields Avenue, Davis, CA 95616. Ph.D. student

Olivier Larouche, Department of Biological Sciences, Clemson University, Clemson SC 29634, USA. Postdoctoral Scholar

Anna J. Lee, Department of Evolution & Ecology, University of California Davis, 1 Shields Avenue, Davis, CA 95616. Undergraduate Student

Kasey Brockelsby, Department of Evolution & Ecology, University of California Davis, 1 Shields Avenue, Davis, CA 95616. Undergraduate Student

Maya Nagaraj, Department of Evolution & Ecology, University of California Davis, 1 Shields Avenue, Davis, CA 95616. Undergraduate Student

Katerina Zapfe, Department of Biological Sciences, Clemson University, Clemson SC 29634, USA. Masters Student

Maxwell F. Rupp, Department of Evolution & Ecology, University of California Davis, 1 Shields Avenue, Davis, CA 95616. Technician

Nick G. Betrand, Department of Evolution & Ecology, University of California Davis, 1 Shields Avenue, Davis, CA 95616. Technician

Angelly Tovar, Department of Evolution & Ecology, University of California Davis, 1 Shields Avenue, Davis, CA 95616. Undergraduate Student

Victoria Susman, Department of Evolution & Ecology, University of California Davis, 1 Shields Avenue, Davis, CA 95616. Undergraduate Student

Jennifer A. Nguyen, Department of Evolution & Ecology, University of California Davis, 1 Shields Avenue, Davis, CA 95616. Undergraduate Student

Rachel Friedman, unaffiliated.

Laura L.J. Vary, Department of Evolution & Ecology, University of California Davis, 1 Shields Avenue, Davis, CA 95616. Undergraduate Student

John R. Estrada, Department of Evolution & Ecology, University of California Davis, 1 Shields Avenue, Davis, CA 95616. Undergraduate Student

Monica Linares, Department of Evolution & Ecology, University of California Davis, 1 Shields Avenue, Davis, CA 95616. Undergraduate Student

Brian Landry, Department of Evolution & Ecology, University of California Davis, 1 Shields Avenue, Davis, CA 95616. Undergraduate Student

Dominique Gross, Department of Biological Sciences, Clemson University, Clemson SC 29634, USA. Undergraduate Student

Mikayla Iwan, Department of Biological Sciences, Clemson University, Clemson SC 29634, USA. Undergraduate Student

Megan Coyne, Department of Evolution & Ecology, University of California Davis, 1 Shields Avenue, Davis, CA 95616. Undergraduate Student

Allison Proffitt, Department of Evolution & Ecology, University of California Davis, 1 Shields Avenue, Davis, CA 95616. Undergraduate Student

Evan Hoeft, Department of Evolution & Ecology, University of California Davis, 1 Shields Avenue, Davis, CA 95616. Undergraduate Student

Erin Shen, Department of Evolution & Ecology, University of California Davis, 1 Shields Avenue, Davis, CA 95616. Undergraduate Student

Mailee Danao, Department of Evolution & Ecology, University of California Davis, 1 Shields Avenue, Davis, CA 95616. Undergraduate Student

Jo Hsuan Kao, Department of Evolution & Ecology, University of California Davis, 1 Shields Avenue, Davis, CA 95616. Undergraduate Student

Sierra Rodriguez, Department of Biological Sciences, Clemson University, Clemson SC 29634, USA. Undergraduate Student

Carley McGlinn, Department of Biological Sciences, Clemson University, Clemson SC 29634, USA. Undergraduate Student

Class III. Data set status and accessibility

A. Status

1. **Latest update:** Headings were updated November 16th 2021 and data June 12th 2019
2. **Latest archive date:** Not applicable
3. **Metadata status:** Metadata updated as of June 20th 2022
4. **Data verification:** Data were cleaned, checked and verified by STF & SAP using the steps outlined in Class V Section B.

B. Accessibility

1. **Storage location and medium:** Data_S1.zip with this publication and Dryad online data repository https://doi.org/10.5061/dryad.vt4b8gtvf
2. **Contact person(s):** Samantha A. Price, Department of Biological Sciences, Clemson University, Clemson SC 29634, USA. Email: sprice6@clemson.edu
3. **Copyright restrictions:** This work is licensed under a Creative Commons CC0 1.0 Universal (CC0 1.0). We request whenever these data or a subset of it are used that this publication in Ecology is cited.
4. **Proprietary restrictions:** None

C. Costs: None

Class IV. Data structural descriptors

A. Data set file

1. **Identity:**
   a. FishShapes_specimensv1.csv
2. **Size:**
   a. 16524 records (including header) and 13 fields. Total file size is 1.7 MB
3. **Header information:** The first row of the data file contains variable names and each row represents data for a single specimen in a. FishShapes_specimensv1.csv
4. **Alphanumeric attributes:** mixed
5. **Special characters/fields**: missing fields are coded as NA
6. **Authentication procedures:**
   a. FishShapes_specimensv1.csv SHA-256 checksum 7447fdc92c5fd5c9ecf1d9713bbd4207d2de7ab4b2ea0e320871041da8e34264

B. Variable information

Dataset. FishShapes_specimensv1.csv

Variable identity: SpecimenID

Variable definition: The catalog number of the specimen. If no museum prefix is provided it is from the Smithsonian National Museum of Natural History collections (USNM). If more than one specimen was measured from the same jar the catalog number was appended with an underscore and a number 1 to 3, the exact specimens can then be identified from their standard length and other measurements. If the specimen is uncatalogued any useful information from the label that might be used to identify the specimen was added in this field otherwise it was listed as UNCAT.

Units of measurement: not applicable

Data type:

a. *Storage type*: string
b. *Range for numeric values*: not applicable
c. *Missing value codes*: UNCAT
d. *Precision*: not applicable

Data format:

a. Variable length
b. Column(s) A

Variable identity: Tree_name

Variable definition: The Latin binomial of the species given in the subset of the Rabosky et al. 2018 phylogeny with molecular data. If the species was not present in that phylogeny the Latin binomial written on the jar in the collection.

Units of measurement*:* not applicable

Data type:

a. *Storage type:* string
b. *Range for numeric values:* not applicable
c. *Missing value codes:* NA
d. *Precision:* not applicable

Data format:

a. Variable length
b. Column(s) B

Variable identity: Family

Variable definition: Family name of the species according to the FishBase taxonomy, as checked on February 20th 2019.

Units of measurement: not applicable

Data type:

a. *Storage type*: string
b. *Range for numeric values*: not applicable
c. *Missing value codes*: NA
d. *Precision*: not applicable

Data format:

a. Variable length
b. Column(s) C

Variable identity: Order

Variable definition: Order name of the species according to the phylogenetic classification of bony fishes v3 (Betancur-R et al. 2014).
Units of measurement: not applicable
Data type:

a. *Storage type*: string
b. *Range for numeric values*: not applicable
c. *Missing value codes*: NA
d. *Precision*: not applicable

Data format:

a. Variable length
b. Column(s) D

Variable identity: Total_weight

Variable definition: specimen weight
Units of measurement: grams
Data type:

a. *Storage type*: numeric - floating point
b. *Range for numeric values*: 0.03 - 39900
c. *Missing value codes*: NA
d. *Precision*: between 2 and 6 significant digits

Data format:

a. Variable length
b. Column(s) E
c. Number of decimal places: 2

Variable identity: Standard_length

Variable definition: specimen length, measured as the straight-line distance from the most anterior tip of the upper jaw to the mid-lateral posterior edge of the hypural plate (in fishes with a hypural plate), or to the posterior end of the vertebral column in fishes lacking them (i.e., excluding the caudal fin). This was identified by manipulating the specimen and looking for a wrinkle on the caudal peduncle when the caudal region is flexed.
Units of measurement: millimeters
Data type:

a. *Storage type*: numeric - floating point
b. *Range for numeric values*: 10.32 - 1760
c. *Missing value codes:* NA
d. *Precision*: between 4 and 6 significant digits

Data format:

a. Variable length
b. Column(s) F
c. Number of decimal places: 2

Variable identity: Max_body_depth

Variable definition: specimen maximum body depth, measured as the greatest depth of the straight-line distance from dorsal to ventral surface of the body, with

body defined as the region posterior to the operculum and anterior to the caudal peduncle.
Units of measurement: millimeters
Data type:
a. *Storage type*: numeric – floating point
b. *Range for numeric values*: 0.98 – 590
c. *Missing value codes*: NA
d. *Precision*: between 2 and 5 significant digits

Data format:
a. Variable length
b. Column(s) G
c. Number of decimal places: 2

Variable identity: Max_fish_width
Variable definition: specimen maximum width, measured as the width of the fish taken at its maximum anywhere on the specimen.
Units of measurement: millimeters
Data type:
a. *Storage type*: numeric – floating point
b. *Range for numeric values*: 1.2 – 184.17
c. *Missing value codes*: NA
d. *Precision*: between 2 and 5 significant digits

Data format:
a. Variable length
b. Column(s) H
c. Number of decimal places: 2

Variable identity: Head_depth
Variable definition: specimen head depth, measured as the vertical distance from dorsal to ventral surface of the head passing through the pupil of the eye.
Units of measurement: millimeters
Data type:
a. *Storage type*: numeric – floating point
b. *Range for numeric values*: 0.71 – 420
c. *Missing value codes*: NA
d. *Precision*: between 2 and 5 significant digits

Data format:
a. Variable length
b. Column(s) I
c. Number of decimal places: 2

Variable identity: Lower_jaw_length
Variable definition: specimen lower jaw length, measured as the length of the mandible from the anterior end of the lower jaw to the articular–quadrate joint. The articular–quadrate joint was identified by feeling for the joint, along with moving the lower jaw and identifying the point where the movement stopped. Occasionally, when the jaw would not move, we estimated the position of the joint by inferring it from the end of the opercular slit or by feeling for the

preopercle bone and run anteriorly along it to its end. The method used to identify the joint was noted in the data entry spreadsheet. Due to identifying the articular-quadrate joint, this was one of most difficult traits to measure.
Units of measurement*:* millimeters
Data type:
a. *Storage type*: numeric – floating point
b. *Range for numeric values*: 0.5 – 292.5
c. *Missing value codes*: NA
d. *Precision*: between 1 and 5 significant digits

Data format:
a. Variable length
b. Column(s) J
c. Number of decimal places: 2

Variable identity: Mouth_width
Variable definition: specimen mouth width, measured as the width of the fish repesenting the distance between the left and right articular–quadrate joints. The articular–quadrate joint was identified by feeling for the joint, along with moving the lower jaw and identifying the point where the movement stopped. Occasionally, when the jaw would not move, we estimated the position of the joint by inferring it from the end of the opercular slit or by feeling for the preopercle bone and run anteriorly along it to its end. The method used to identify the joint was noted in the data entry spreadsheet. Due to identifying the articular-quadrate joint, this was one of most difficult traits to measure.
Units of measurement*:* millimeters
Data type:
a. *Storage type*: numeric – floating point
b. *Range for numeric values*: 0.27 – 162
c. *Missing value codes*: NA
d. *Precision*: between 1 and 5 significant digits

Data format:
a. Variable length
b. Column(s) K
c. Number of decimal places: 2

Variable identity: Min_caudalpeduncle_depth
Variable definition: specimen minimum caudal peduncle depth, measured as the depth measured by a straight-line distance from dorsal to ventral surface of the caudal peduncle at its shallowest point. The anterior extremity of the caudal peduncle region was defined as the most posterior reaching of the two median fins (dorsal or anal fin) and the posterior extremity by caudal fin base.
Units of measurement: millimeters
Data type:
a. *Storage type*: numeric – floating point
b. *Range for numeric values*: 0.15 – 84.37
c. *Missing value codes*: NA
d. *Precision*: between 1 and 4 significant digits

Data format:

a. Variable length
b. Column(s) L
c. Number of decimal places: 2

Variable identity: Min_caudalpeduncle_width

Variable definition: specimen minimum caudal peduncle width, measured as the width of the fish measured at its narrowest point on of the caudal peduncle. The anterior extremity of the caudal peduncle region was defined as the most posterior reaching of the two median fins (dorsal or anal fin) and the posterior extremity by caudal fin base.
Units of measurement: millimeters
Data type:
a. *Storage type*: numeric – floating point
b. *Range for numeric values*: 0.03 – 58.69
c. *Missing value codes*: NA
d. *Precision*: between 1 and 4 significant digits

Data format:
a. Variable length
b. Column(s) M
c. Number of decimal places: 2

C. Data anomalies:

Missing data. Within the specimen dataset FishShapes_specimensv1.csv the percentage of missing data per trait ranges between 0.05% (8/16523) for standard length to 0.59% (97/16523) for total weight. Missing data for total weight is primarily due to specimens exceeding the minimum or maximum weight of our scales. Missing data for the other traits is primarily due to specimen damage that prevented us from measuring that trait e.g. missing jaws or caudal region. A few missing values were added during our quality assurance procedures when we could not be certain of the data entered, as explained in Class V section B.

Total weight. The total weight of the specimen is influenced by several factors including preservation, specimen damage (e.g. some specimens had been dissected with some or all of their internal organs removed) and metal tags attached to the specimen. We therefore suggest anyone planning to use total weight carefully consider these issues before use.

Class V. Supplemental descriptors

A. Data acquisition

1. **Data forms or acquisition methods:** see Class II, Section B.
2. **Location of completed data forms:** data entry forms are stored in the cloud (Clemson University Google Drive) with an offline backup on an external hard drive).
3. **Data entry verification procedures:** Several steps taken to ensure that the data entered were error free. First, prior to going to the museum all personnel responsible for measuring specimens were carefully trained to recognize and

measure each trait on a broad taxonomic sample of fishes and to ask for advice if unsure (see Price et al. 2020). Following training the students were given a test dataset measured by the senior personnel and those with the most accurate measurements (closest to the values measured by two or more senior personnel) were invited to join the data collection team. Second, while at the museum data collectors were reminded to: ensure their calipers were frequently zeroed, if they were uncertain about anything ask for advice and if they thought they may have made a mistake to let someone know so we could check and correct it (see Read Me.pdf).

B. **Quality assurance/quality control procedures**: We used a three-step process to identify potential outliers, first comparing specimens within a species, second specimens within a genus and finally specimens within a family. All outliers were added to our trait check document for manual checking.

Step I: Species-level checks

1. **Species name and catalog number check**: As we were only measuring a maximum of three specimens per species, we first determined which species had more than three specimens in the dataset and then we checked the USNM catalog numbers of those with >3 specimens against the online Smithsonian database to ensure they corresponded to the correct species name.
2. **Automatic trait check**
    - All traits were first size corrected by dividing by the specimen's standard length measurement. We then computed standard deviations of all traits within each species and filtered by a set cut off for the minimum acceptable within-species standard deviation of 0.1 for all traits except body mass, which had a cut-off of 3, due to the large variation in specimen size. All measurements that exceeded the cut-off were added to the trait document to be checked manually. Species with a single specimen in our dataset were also added to the trait check document for manual checking, as were specimens where the recorded max body width was less than the reported mouth width.

Step II: Genus-level checks

1. **Automatic trait check**
    - All length, width and depth traits were first size corrected by dividing by the specimen's standard length measurement
    - We created a function to locate outlier specimens for each trait relative to all specimens in the genus, the specimen ID and the trait were added to our trait check document to be inspected manually. Outliers were defined as follows: x < quantile(x, 0.05) - 1.5*IQR(x) | x > quantile(x, 0.95) + 1.5*IQR(x). This means specimens were classified as outliers if the value is less than 1.5 times the interquartile range (IQR) subtracted from the 5th percentile or if the value is greater than 1.5 times the interquartile range added to the 95th percentile. For a given trait, if all three specimens of a species were listed as outliers, these were

considered normal morphological variation and thus not added to the trait check document.

Step III: Family-level checks

1. **Taxon check**: Family classifications in our data set were checked against family classifications in FishBase for each species. Those that disagreed were manually checked and corrected. We added all specimens that were the only species in their family to the trait check document and manually searched for erroneous measurements.
2. **Automatic trait check:** This step had the same filtration criteria as the genus-level check, but instead of looking for outlier specimens for each trait relative to its genus, we found outlying specimens relative to the family as a whole. These specimens were added to the trait check document to be manually inspected. At each step, boxplots were used to visualize the extent of outlier specimens within families to confirm that the function was adequately capturing problematic specimens.

Manual trait check

All specimen measurements that were listed in the trait check document were manually checked by STF & SAP. Exceptionally large or small values were checked to see if they were potentially caused by the misplacement of the decimal point during data entry. We compared the values with the decimal point changed to see if the values matched those of the other specimens and of the other traits measured on that specimen. Additionally, length and depth measurements were compared to the lateral scaled photograph of the specimen to see if the values were within those estimated from the photograph. Any outlying value that we could not confidently identify as either a misplacement of decimal place or a correct estimate based on the photograph was removed from the dataset.

C. **Related materials:** All specimens measured were in the Smithsonian National Museum of Natural History fish collections.

D. **Computer programs and data-processing algorithms:** Trait data were checked and cleaned using the statistical computing framework R v3.5 (R Development Core Team 2019) and the packages tidyverse (Wickham et al. 2019) and rfishbase (Boettiger et al. 2012).

E. **Archiving**

1. **Archival procedures:** All previous versions, intermediate datasets generated during cleaning and verification along with the original data forms are archived and stored in the cloud as .csv and .zip files (Clemson University google drive) as well as offline on an external hard drive.

2. **Redundant archival sites:** Not applicable

F. **Publications and results:**

Body width is the primary axis of shape variation within this dataset once phylogeny and size (using log-shape ratios) have been incorporated: dorsoventrally flattened fishes are

at one extreme and laterally compressed fishes at the other (Price et al. 2019). The secondary axis is elongation, contrasting long, slender and narrow fishes with short, deep and wide fishes. We note these axes change if different methods of size correction are used and if phylogeny is ignored (Price et al. 2019). When including ecological data our results so far reveal that habitat and environmental conditions likely impose strong selective pressures, driving consistent patterns of fish body shape evolution and diversification on a global scale. Analyses of our dataset have found support at the macroevolutionary scale for long-standing predictions based on mechanical models concerning the impact of structurally complex environments (Larouche et al., 2020) and extreme depths on fish body shape (Martinez et al., 2021). Similarly, our research has revealed body shape changes along the benthic-pelagic axis across marine and freshwater teleosts that are consistent with studies of ecological diversification within specific lineages (Friedman et al., 2020, Friedman, Collyer et al., 2021). Other influencing body shape include the mode of swimming used by fishes (Friedman, Price & Wainwright 2021) and their feeding mode (Corn et al. In Review). In addition, our results have also highlighted the complexity of shape evolution, identifying lineage-specific effects (e.g. Price et al., 2019, Alencar et al., 2022) and divergent processes that drive similar patterns (Friedman, Collyer et al., 2021).

Price, S. A., Friedman, S.T., Corn, K.A., Martinez, C.M., Larouche, O. & Wainwright, P.C. (2019) Building a body shape morphospace of teleostean fishes. *Integrative and Comparative Biology* 59(3), 716-730.

Price, S. A., Larouche, O., Friedman, S.T., Corn, K.A. & Wainwright, P.C & Martinez, C.M. (2020) A CURE for a major challenge in phenomics: a practical guide to implementing a quantitative specimen-based undergraduate research experience. *Integrative Organismal Biology* 2(1), p.obaa004.

Friedman, S.T., Price, S.A., Corn, K.A., Larouche, O., Martinez, C.M. and Wainwright, P.C. (2020) Body shape diversification along the benthic-pelagic axis in marine fishes. *Proceedings of the Royal Society of London, B.* 287(1931), 20201053

Larouche, O.[*], Benton, B., Corn, K.A., Friedman, S.T., Gross, D., Iwan, M., Kessler, B., Martinez, C.M., Rodriquez, S., Whelpley, H., Wainwright, P.C. & Price, S.A. (2020) Reef-associated fishes have more manoeuvrable body shapes at a macroevolutionary scale. (2020) *Coral Reefs* *39*(5), pp.1427-1439

Larouche, O., Alencar, Laura R. V., Hodge, J.R., Camper, B., Adams, D. S., Zapfe, K.L., Friedman, S. T., Wainwright, P. C., and Price, S. A. (2020) Do key innovations unlock diversification? A case-study on the morphological and ecological impact of pharyngognathy in acanthomorph fishes. *Current Zoology* *66*(5), pp.575-588

Martinez, C.M., Friedman, S.T., Corn, K.A., Larouche, O., Price, S.A. & Wainwright, P.C. (2021) The Deep Sea is a Hot Spot of Fish Body Shape Evolution. *Ecology Letters* 24(9), 1788-1799

Friedman, S. T., Price, S.A. & Wainwright P.C. (2021) The effect of locomotion mode on body shape evolution in teleost fishes. (2021) *Integrative Organismal Biology 3(1),* obab016

Friedman, S.T., Collyer, M.L., Price, S.A. & Wainwright, P.C. (2021) Divergent processes drive parallel evolution in marine and freshwater fishes, *Systematic Biology*, syab080, https://doi.org/10.1093/sysbio/syab080

Alencar, L.R.V, Hodge, J. R., Friedman, S.T., Wainwright, P.C. & Price, S.A. (2022) Size as a complex trait and the scaling relationships of its components across teleost fishes. Evolutionary Ecology, https://doi.org/10.1007/s10682-022-10177-6

Corn, K.A., Friedman, S.T., Burress, E.D., Martinez, C.M., Larouche, O., Price, S.A. & Wainwright, P.C. (revised 3/3/2022) The trophic revolution among reef fishes in the Cenozoic. Proceedings of the National Academy of Sciences

G. **History of data set usage:** Not applicable

REFERENCES

Andersson, J. (2003). Effects of diet-induced resource polymorphism on performance in Arctic charr (*Salvelinus alpinus*). *Journal of Evolutionary Ecological Research* 5, 213–28.

Bainbridge R. (1960). Speed and stamina in three fish. *Journal of Experimental Biology* 37,129–153

Betancur-R, R., E. Wiley, N. Bailly, M. Miya, G. Lecointre, and G. Ortí. 2014. Phylogenetic classification of bony fishes --Version 3 (http://www.deepfin.org/Classification_v3.htm).

Boettiger C, Temple Lang D, Wainwright P (2012). "rfishbase: exploring, manipulating and visualizing FishBase data from R." *Journal of Fish Biology* 81(6), 2030-2039. https://doi.org/10.1111/j.1095-8649.2012.03464.x

Brönmark C. and Miner J.G. (1992). Predator-induced phenotypical change in body morphology in crucian carp. *Science* 258,1348–1350.

Chivers, D.P., Zhao, X., Brown, G.E., Marchant, T.A. and Ferrari, M.C. (2008). Predator-induced changes in morphology of a prey fish: the effects of food level and temporal frequency of predation risk. *Evolutionary Ecology* 22(4), 561-574.

Collar D.C., Near T.J. and Wainwright P.C. (2005). Comparative analysis of morphological diversity: trophic evolution in centrarchid fishes. *Evolution* 59, 1783 -1794.

Domenici, P. (2003). Habitat, body design and the swimming performance of fish, in Vertebrate biomechanics and evolution V.L. Bels, A. Casinos, and J.-P. Gasc, Editors. 2003, Society for Experimental Biology, 137-60.

Domenici P. (2010). Escape responses in fish: kinematics, performance and behavior. In: Domenici P, Kapoor BG (eds) Fish locomotion: an eco-ethological perspective. Science Publishers, Enfield, 123–170

Felsenstein, J. (1985). Phylogenies and the comparative method. *The American Naturalist* 125(1), 1-15.

Friedman, S.T., Price, S.A., Corn, K.A., Larouche, O., Martinez, C.M. and Wainwright, P.C. (2020) Body shape diversification along the benthic-pelagic axis in marine fishes. *Proceedings of the Royal Society of London, B.* 287(1931), 20201053

Froese, R. and D. Pauly. Editors. 2019. FishBase. World Wide Web electronic publication. www.fishbase.org

Gatz, A. J. 1979. Ecological morphology of freshwater stream fishes. *Tulane Studies in Zoology and Botany* 21, 91-124.

Hambright, K.D., 1991. Experimental analysis of prey selection by largemouth bass: role of predator mouth width and prey body depth. *Transactions of the American Fisheries Society* 120(4), 500-508.

Helfman, G.S., Collette, B.B., Facey, D.E., and Bowen, B.W. (2009). The Diversity of Fishes. Biology, Evolution & Ecology. Oxford: Wiley-Blackwell.

Langerhans, R.B., Chapman, L.J., and Dewitt, T.J. (2007). Complex phenotype environment associations revealed in an East African cyprinid. *Journal of Evolutionary Biology* 20, 1171-81.

Langerhans, R.B. and Reznick D.N. (2010). Ecology and evolution of swimming performance in fishes: predicting evolution with biomechanics. In: Domenici P, Kapoor BG (eds) Fish locomotion: an eco-ethological perspective. Science Publishers, Enfield, pp 200–248

Lauder, G.V. (1980) The suction feeding mechanism in sunfishes (Lepomis): an experimental analysis. *Journal of Experimental Biology* 88, 49–72.

Martin, W.R. (1949) The mechanics of environmental control of body form in fishes. Publications of the Ontario Fisheries Research Laboratory Vol. 70. Toronto: University of Toronto Press.

Oliveira, E. F., E. Goulart, L. Breda, C. V. Minte-Vera, L. R. S. Paiva & M. R. Vismara. (2010). Ecomorphological patterns of the fish assemblage in a tropical floodplain: effects of trophic, spatial and phylogenetic structures. *Neotropical Ichthyology* 8, 569-586.

Price, S. A., Larouche, O., Friedman, S.T., Corn, K.A. & Wainwright, P.C & Martinez, C.M. (2020) A CURE for a major challenge in phenomics: a practical guide to implementing a quantitative specimen-based undergraduate research experience. *Integrative Organismal Biology 2*(1), p.obaa004.

R Core Team 2019. R: A language and environment for statistical computing. R Foundation for Statistical Computing, Vienna, Austria. URL https://www.R-project.org/.

Rabosky, D.L., Chang, J., Title, P.O., Cowman, P.F., Sallan, L., Friedman, M., Kaschner, K., Garilao, C., Near, T.J., Coll, M. and Alfaro, M.E. (2018) An inverse latitudinal gradient in speciation rate for marine fishes. *Nature*, *559*(7714), 392-395.

Rand, D.M. and Lauder, G.V. (1981). Prey capture in the chain pickerel, *Esox niger*: correlations between feeding and locomotor behavior. *Canadian Journal of Zoology* 59, 1072-78.

Robinson, B.W. and Parson, K.J. (2002). Changing times, spaces, and faces: tests and implications of adaptive morphological plasticity in the fishes of northern postglacial lakes. *Canadian Journal of Fisheries and Aquatic Science* 59, 1819-33.

Norton, S. F. & E. L. Brainerd. (1993). Convergence in the feeding mechanics of ecomorphologically similar species in the Centrarchidae and Cichlidae. *Journal of Experimental Biology* 176, 11-29.

Sweet, J.G. and Kinne, O. (1964). The effects of various temperature-salinity combinations on the body form of newly hatched *Cyprinodon macularius* (Teleostei). *Helgoländer wissenschaftliche Meeresuntersuchungen* 11(2), 49-69.

Wainwright, P.C. and Richard, B.A. (1995). Predicting patterns of prey use from morphology of fishes. *Environmental biology of fishes*, *44*(1), 97-113.

Wainwright, P.C., Carroll, A.M., Collar, D.C., Day, S.W., Higham, T.E., and Holzman, R.A. (2007). Suction feeding mechanics, performance, and diversity in fishes. *Integrative and comparative biology* 47(1), 96-106.

Walker J.A., Alfaro M.E., Noble M.M. and Fulton C.J. (2013) Body fineness ratio as a predictor of maximum prolonged-swimming speed in coral reef fishes. *PLoS One* 8:e75422

Webb P.W. (1984). Body form, locomotion and foraging in aquatic vertebrates. *American Zoologist* 24,107–120

Webb P.W. (1994). The biology of fish swimming. In: Maddock L, Bone Q, Rayner JMV (eds) Mechanics and physiology of animal swimming. Cambridge University Press, Cambridge, 45–62

Weihs, D. (1973). The mechanism of rapid starting of slender fish. *Biorheology* 10, 343-50.

Westneat, M.W. (2004). Evolution of levers and linkages in the feeding mechanisms of fishes. *Integrative and Comparative Biology 44*(5), 378-389.

Wickham H, Averick M, Bryan J, Chang W, McGowan LD, François R, Grolemund G, Hayes A, Henry L, Hester J, Kuhn M, Pedersen TL, Miller E, Bache SM, Müller K, Ooms J, Robinson D, Seidel DP, Spinu V, Takahashi K, Vaughan D, Wilke C, Woo K, Yutani H (2019). “Welcome to the tidyverse.” *Journal of Open Source Software*, **4**(43), 1686. doi: https://doi.org/10.21105/joss.01686.
